# Evaluation of six TPS algorithms in computing entrance and exit doses

**DOI:** 10.1120/jacmp.v15i3.4739

**Published:** 2014-05-08

**Authors:** Yun I. Tan, Mohamed Metwaly, Martin Glegg, Shaun P. Baggarley, Alex Elliott

**Affiliations:** ^1^ College of Medical Veterinary, and Life Sciences, University of Glasgow Scotland UK; ^2^ Radiotherapy Centre National University Cancer Institute Singapore; ^3^ Radiotherapy Physics The Beatson West of Scotland Cancer Centre Scotland UK

**Keywords:** *in vivo* dosimetry, entrance and exit dose, TPS algorithms

## Abstract

Entrance and exit doses are commonly measured in *in vivo* dosimetry for comparison with expected values, usually generated by the treatment planning system (TPS), to verify accuracy of treatment delivery. This report aims to evaluate the accuracy of six TPS algorithms in computing entrance and exit doses for a 6 MV beam. The algorithms tested were: pencil beam convolution (Eclipse PBC), analytical anisotropic algorithm (Eclipse AAA), AcurosXB (Eclipse AXB), FFT convolution (XiO Convolution), multigrid superposition (XiO Superposition), and Monte Carlo photon (Monaco MC). Measurements with ionization chamber (IC) and diode detector in water phantoms were used as a reference. Comparisons were done in terms of central axis point dose, 1D relative profiles, and 2D absolute gamma analysis. Entrance doses computed by all TPS algorithms agreed to within 2% of the measured values. Exit doses computed by XiO Convolution, XiO Superposition, Eclipse AXB, and Monaco MC agreed with the IC measured doses to within 2%‐3%. Meanwhile, Eclipse PBC and Eclipse AAA computed exit doses were higher than the IC measured doses by up to 5.3% and 4.8%, respectively. Both algorithms assume that full backscatter exists even at the exit level, leading to an overestimation of exit doses. Despite good agreements at the central axis for Eclipse AXB and Monaco MC, 1D relative comparisons showed profiles mismatched at depths beyond 11.5 cm. Overall, the 2D absolute gamma (3%/3 mm) pass rates were better for Monaco MC, while Eclipse AXB failed mostly at the outer 20% of the field area. The findings of this study serve as a useful baseline for the implementation of entrance and exit *in vivo* dosimetry in clinical departments utilizing any of these six common TPS algorithms for reference comparison.

PACS numbers: 87.55.‐x, 87.55.D‐, 87.55.N‐, 87.53.Bn

## I. INTRODUCTION

*In vivo* dosimetry in radiation therapy is recommended to prevent major errors in treatment delivery.^1^, ^2^, ^3^, ^4^, ^5^ *In vivo* dosimetry can be performed either in real time or passively using different types of detectors.^2^, ^6^, ^7^ Ideally, detectors should be placed close to the organ of interest, but most *in vivo* measurements are done at the beam entrance, exit, or both, to avoid invasive application. In the case where an electronic portal imaging device is used as the detector, measured dose can be back‐projected to the entrance or exit level of the patient.^8^, ^9^ Measured or back‐projected doses at the entrance and exit level are compared to an expected range, usually generated by the treatment planning system (TPS), to verify accuracy of treatment delivery.^(9)^

When using the TPS as a reference for dose verification, it is essential that the TPS calculates doses at the entrance and exit level for all conditions in the clinical setting to a high degree of accuracy. However, due to the limited buildup and backscatter at the entrance and exit levels, respectively, the accuracy of TPS dose computation must be validated. Although accuracy of different TPS algorithms had been extensively reported, these studies mainly focused on dose nearer to the isocenter and with the presence of inhomogeneity.^10^, ^11^, ^12^, ^13^, ^14^, ^15^, ^16^, ^17^, ^18^

The aim of this report was to perform a fundamental verification of six commercial TPS algorithms in computing entrance and exit doses for a 6 MV beam. Ionization chamber (IC) and diode detector measurements were used as a reference. Entrance and exit doses were defined as doses at depths of 1.5 cm from the beam entry and exit surfaces, respectively. This depth of 1.5 cm was chosen to provide reasonable electronic equilibrium condition for the 6 MV beam investigated. Dose uncertainties in the buildup region were beyond the scope of this study and can be found in other publications.^19^, ^20^, ^21^, ^22^, ^23^, ^24^, ^25^, ^26^, ^27^ The same depth for exit dose was chosen for a symmetric geometry.

Central axis point dose, 1D relative profiles, and 2D absolute dose comparisons were investigated for a range of clinically relevant field sizes and physical thicknesses in homogeneous water phantoms. Inhomogeneous phantoms were not considered. 1D and 2D comparisons were included in this study to provide additional information on off‐axis accuracies, which are important for 2D *in vivo* dosimetry (for example, 2D EPID dosimetry).

## II. MATERIALS AND METHODS

### A. Central axis point dose analysis

#### A.1 TPS virtual simulation

**Figure 1:**
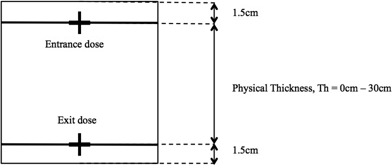
Virtual water phantom with physical thickness (Th) ranging from 0 cm to 30 cm. Entrance and exit doses were defined as doses at 1.5 cm from the beam entry and exit surface, respectively.

Virtual water phantoms with dimensions of 30 cm×30 cm×1.5+Th+1.5 cm (where *Th* ranged from 0 cm to 30 cm) were created in Eclipse, XiO, and Monaco TPSs (Fig. 1). To maintain consistency, the same investigator created the phantoms on all TPSs. The range of physical thickness, Th, was chosen to include all clinically relevant thicknesses. Entrance and exit doses, defined as doses at 1.5 cm from the beam entry and exit surface, were computed for a 6 MV beam, source‐to‐surface distance (SSD) 100 cm, at gantry zero degree, and field size ranging from $5\;{\text{cm}}^{2}$ to $20\;{\text{cm}}^{2}$ using six different algorithms on three TPSs:

1. Eclipse TPS (version 10.0.28, Varian Medical Systems, Palo Alto, CA,)
  – pencil beam convolution (Eclipse PBC)
  – analytical anisotropic algorithm (Eclipse AAA)
  – AcurosXB (Eclipse AXB)
2. XiO TPS (version 4.70, Elekta AB, Stockholm, Sweden)
  – FFT convolution (XiO Convolution)
  – multigrid superposition (XiO Superposition)
3. Monaco TPS (version 3.20.02, Elekta AB, Stockholm, Sweden)
  – Monte Carlo photon (Monaco MC)

Doses on all TPSs, except for Monaco MC, were calculated using 0.1 cm grid size with the calculation box enclosing the outer border of the virtual phantoms. Monaco MC calculation grid was set to 0.2 cm with 1% standard deviation per plan criteria. Larger calculation grid size was set for Monaco MC because of hardware limitation.

The TPSs were located in two separate and independent institutions: Institution A (Inst. A) is equipped with the Eclipse TPS commissioned for Varian Clinac 21EX (Palo Alto, CA) linear accelerator, while Institution B (Inst. B) is equipped with XiO and Monaco TPSs commissioned for Elekta Synergy (Stockholm, Sweden) linear accelerator. All comparisons described in this study were performed between TPS and the corresponding linear accelerator for which the TPS was commissioned.

#### A.2 Experimental measurement

Using a 0.6 cc Farmer IC (Inst. A: NE2581, Nuclear Enterprises, Fairfield, NJ and Inst. B: PTW30001, Freiburg, Germany) in water‐equivalent solid phantoms of dimension 30 cm×30 cm (Inst. A: Solid Water with density $1.04g/{\text{cm}}^{3}$, Gammex Inc., Middleton, WI, and Inst. B: Plastic Water with density $1.03g/{\text{cm}}^{3}$, CIRS Inc., Norfolk, VA) set up at SSD 100 cm, entrance and exit doses were measured for a 6 MV beam on the Clinac 21EX and Synergy linear accelerators. Solid phantoms were positioned flat on treatment couch to ensure stability and reproducibility of setup. Entrance doses were measured at depth 1.5 cm, with backscatter thickness ranging from 1.5 cm to 31.5 cm while exit doses were measured at depths 1.5 cm to 31.5 cm with backscatter thickness of 1.5 cm. Doses were measured at gantry zero degree for field size $5\;{\text{cm}}^{2}$ to $20\;{\text{cm}}^{2}$. This experimental setup replicates the condition described in Materials and Methods section A.1 above for TPS dose computations. To avoid uncertainties related to differences in chambers, electrometers, and water‐equivalent solid phantoms in the two institutions, a ‘relative’ approach was taken. As outputs for linear accelerators at both institutions were calibrated in water to 1cGy/MU at calibration condition, readings were first taken in charge mode at calibration condition to obtain the charge to dose conversion factor. Subsequent measurements were measured in charge and converted to dose using this factor. This method also allowed variation in daily output to be corrected. In addition, measurements were repeated with a CC04 IC (IBA Dosimetry, Schwarzenbruck, Germany) to provide a crosscheck on the accuracy of the data measured with the 0.6 cc Farmer IC, especially for the smallest field size of $5\;{\text{cm}}^{2}$. To maintain consistency, the same investigator performed all measurements in both institutions. Doses measured on the linear accelerators were compared with TPS computed doses.

#### A.3 Deriving backscatter correction factor (BCF)

Backscatter correction factor, BCF, was defined as the ratio of dose without backscatter to dose with full backscatter (Eq. (1)).

(1) $$\text{Backscatter Correction Factor},\text{BCF}=\frac{\text{Dose without backscatter}}{\text{Dose with full backscatter}},\;\text{where}\;\text{BCF}\;\text{is}\;\leq 1$$

Doses without backscatter, also used interchangeably as exit doses in this study, were the doses measured using methods described in Material and Methods section A.2. Doses with full backscatter were calculated using percentage depth dose (PDD) and field size output factor (OF), according to Eq. (2). PDD and OF were measured with CC13 IC (IBA Dosimetry) in a scanning water tank (IBA Dosimetry).

(2) $${\text{Dose}}_{\text{fs}}^{d}=D_{\text{dmax}}\times {\text{PDD}}_{fs}^{d}\times {\text{OF}}_{\text{fs}}$$

where *d* is the depth where dose is calculated, *fs* is the field size at distance 100 cm, is the dose at depth of maximum for reference field size (usually $10\times 10\;{\text{cm}}^{2}$), ${\text{PDD}}_{\text{fs}}^{u}$ is the percentage depth dose, and ${\text{OF}}_{\text{fs}}$ is the field size output factor.

### B. 1D relative profile analysis

To check the accuracies of TPS algorithms in computing off‐axis doses, TPS profiles were compared with profiles measured in a scanning water tank using CC04 IC (IBA Dosimetry) in Inst. A and diode detector (PFD Photon, IBA Dosimetry) in Inst. B. The resolutions of scanned profiles were 0.2 cm in both institutions. The 1D TPS profiles were extracted from 2D entrance and exit dose planes generated from the six TPS algorithms for a 6 MV beam with different field sizes ($5\;{\text{cm}}^{2}$ to $20\;{\text{cm}}^{2}$) at different depths (1.5 cm to 31.5 cm). Meanwhile, IC profiles were not measured at the exit plane because of limitation in measuring with 1.5 cm backscatter using the scanning water tank. The differences in setup between TPS and IC profiles were not critical as backscatter thickness only affects the relative profiles minimally.^(28)^ However, since the IC measured profiles were used as a reference, they were independently checked against exit measurements performed with commercial 2D array devices, MapCHECK2 (Sun Nuclear, Melbourne, FL) in Inst. A and ${\text{MatriXX}}^{\text{Evolution}}$ (IBA Dosimetry) in Inst. B. Water‐equivalent solid phantoms of appropriate thicknesses were placed on top of the devices to measure 2D exit dose planes at various depths (the inherent thickness of material behind the detectors, less than 3.5 cm, were ignored). Profiles were extracted from the 2D array measured dose planes for comparison with the IC measured profiles. All profiles were normalized to the central axis for relative comparisons. Image processing and analysis were performed using MATLAB (R2011a_Student) software (The MathWorks, Natick, MA).

### C. 2D absolute dose analysis

The 1D profiles (in‐plane or cross‐plane), measured with IC in scanning water tank at each depths and field sizes, were cross‐multiplied to generate 2D dose planes. These 2D dose planes, which were in relative mode, were converted to absolute dose by multiplication with the measured central axis point dose. 2D gamma comparisons were used to compare IC measured 2D absolute dose planes with TPS generated dose planes. Results were given as percentage points that passed gamma criterion of 3%/3 mm.

## III. RESULTS

### A. central axis point dose analysis

#### A.1 TPS vs. IC measured

Repeated entrance and exit measurements using two different chambers, 0.6 cc Farmer and CC04 ICs, showed excellent agreement with difference less than 0.7% for the whole range of field sizes ($5\;{\text{cm}}^{2}$ to $20\;{\text{cm}}^{2}$) and backscatter thicknesses or depths (1.5 cm to 31.5 cm) measured in this study. To account for setup uncertainties, repeated measurements with the same chamber on different occasions showed deviation less than 0.5%. The consistency in these results gave confidence to the measured data.

Comparison of entrance doses between TPS‐computed and IC‐measured doses for different field sizes ($5\;{\text{cm}}^{2}$ to $20\;{\text{cm}}^{2}$) and backscatter thicknesses (1.5 cm to 31.5 cm) showed good agreement with deviation less than 2%. All six of the TPS algorithms accurately computed the entrance doses at depth 1.5 cm for the 6 MV beam.

**Figure 2:**
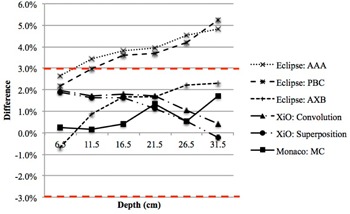
Percentage difference in central axis exit doses between different TPS algorithms and IC measured (only field size $20\;{\text{cm}}^{2}$ is shown). Difference=(TPS computed/IC measured)−1

**Figure 3:**
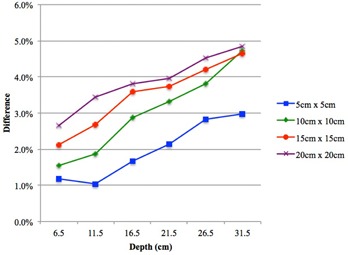
Percentage difference in central axis exit doses between Eclipse AAA and IC measured as a function of depth for different field sizes ($5\;{\text{cm}}^{2}$ to $20\;{\text{cm}}^{2}$). Eclipse AAA computed doses were higher than IC measured doses at the exit level (only Eclipse AAA is shown). Difference=(TPS computed/IC measured)−1

Dose comparisons at the exit level, where the thickness of underlying material was 1.5 cm, showed variable degree of accuracy among the different algorithms (Fig. 2). Exit doses computed with XiO Convolution, XiO Superposition, and Monaco MC agreed with the IC measured doses to within 2.3%. Eclipse AXB showed similar results, except for the smallest field size ($5\;{\text{cm}}^{2}$) where deviation was 3.3%. Poorer results were seen with Eclipse PBC and Eclipse AAA, where the computed exit doses deviated from IC measured doses by up to 5.3% and 4.8%, respectively. In the case of Eclipse PBC and Eclipse AAA, deviations of exit doses increased with increasing field size and depth. Figure 3 shows the percentage exit dose difference between Eclipse AAA with IC measured doses for different field sizes as a function of depth (for clearer illustration, only Eclipse AAA is shown).

#### A.2 Backscatter correction factor (BCF)

To quantify the effect of backscatter thickness on the entrance and exit doses, backscatter correction factor (BCF) was derived as a function of field size and presented in Table 1 for the 6 MV beam from Clinac 21EX and Elekta Synergy linear accelerators. The BCF tables were separated into entrance BCF and exit BCF. Entrance BCF was derived at a fixed depth of 1.5 cm, with underlying material thickness ranging from 1.5 cm to 31.5 cm. Exit BCF was derived at depths ranging from 6.5 cm to 31.5 cm, with the thickness of underlying material being kept constant at 1.5 cm. In the case of entrance BCF, the BCF definition given in Eq. (1) was not strictly accurate as the ratio was taken as ‘dose with partial backscatter to dose with full backscatter’. However, the entrance BCF values were included to provide a realistic clinical representation of entrance dose measurements at different parts of patients' body that have variable backscatter thickness.

**Table 1:** Entrance and exit backscatter correction factor (BCF) for two 6 MV photon beams. Factors were given as a function of field size and thickness of underlying material (backscatter thickness) for the entrance BCF and thickness of overlying material (depth) for the exit BCF

*Entrance BCF (Depth 1.5 cm)*							
*Clinac 21EX* $\left(TPR_{20,10}=00.669\right)$	*Backscatter Thickness (cm)*						
Field Size (${\text{cm}}^{2}$)	1.5	6.5	11.5	16.5	21.5	26.5	31.5
5×5	0.991	0.995	0.998	0.998	0.999	0.999	1.000
10×10	0.985	0.994	0.998	0.999	0.999	1.000	1.000
15×15	0.978	0.991	0.997	0.999	0.999	1.000	1.000
20×20	0.972	0.990	0.996	0.999	0.999	1.001	1.000
*Synergy* ($TPR_{20,10}=0.687$)							
Field Size (${\text{cm}}^{2}$)							
5×5	0.993	0.996	0.997	0.997	0.998	0.998	1.000
10×10	0.987	0.995	0.997	0.998	0.999	0.999	1.000
15×15	0.981	0.993	0.996	0.998	0.999	0.998	1.000
20×20	0.978	0.992	0.996	0.999	1.000	1.000	1.000
*Exit BCF (Backscatter thickness 1.5 cm)*							
*Clinac 21EX* ($TPR_{20,10}=0.669$)	*Depth (cm)*						
Field Size (${\text{cm}}^{2}$)	1.5^a^	6.5	11.5	16.5	21.5	26.5	31.5
5×5	‐	0.995	0.991	0.984	0.976	0.970	0.972
10×10	‐	0.987	0.983	0.980	0.973	0.970	0.960
15×15	‐	0.983	0.978	0.974	0.972	0.968	0.965
20×20	‐	0.981	0.972	0.973	0.969	0.965	0.963
*Synergy* ($TPR_{20,10}=0.687$)							
Field Size (${\text{cm}}^{2}$)							
5×5	‐	1.000	0.993	0.987	0.984	0.977	0.975
10×10	‐	0.988	0.987	0.986	0.985	0.982	0.983
15×15	‐	0.978	0.978	0.976	0.971	0.972	0.971
20×20	‐	0.971	0.968	0.966	0.965	0.962	0.960

a Exit BCF for depth 1.5 cm was equal to entrance BCF with backscatter thickness 1.5 cm and, therefore, only given in the entrance BCF column.

BCF values were very similar for both linear accelerators as the beam quality was almost identical for the 6 MV beam from Clinac (${\text{TPR}}_{20,10}=0.669$) and Synergy (${\text{TPR}}_{20,10}=0.687$). The average differences in BCF values were 0.1%±0.07% and 0.5%±0.47% for the entrance and exit BCF, respectively. Entrance BCF initially increased before it reached unity as the thickness of underlying material and field size increased. The minimum entrance BCF occurred when the underlying material was the smallest, 1.5 cm, and the field size largest, $20\;{\text{cm}}^{2}$. The values were 0.972 for Clinac and 0.978 for Synergy beams. The dose reduction became negligible (less than 0.5% dose reduction) when thickness of underlying material was equal or more than 11.5 cm. Meanwhile, the exit BCF ranged from 0.995 to 0.963 and 1.000 to 0.960 for the Clinac and Synergy beams, respectively. The largest correction occurred at the largest depth and field size.

As most clinical treatments are isocentric with variable SSD, BCF values were also derived for phantoms set up at 100 cm to the center. Comparison of BCF values between fixed SSD 100 cm and variable SSD showed very similar results, with a maximum difference of 0.8% and standard deviation of 0.15% for the whole range of depths and field sizes tested.

### B. 1D relative profile analysis

**Figure 4:**
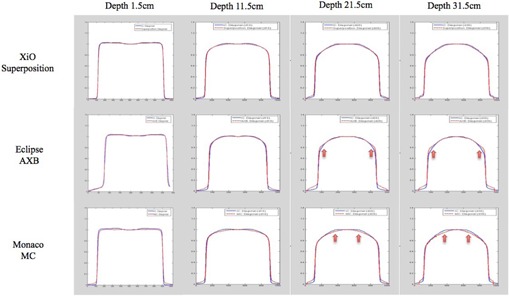
Diagonal relative profiles comparison between TPS computed (red dashed line) and IC measured (blue solid line) at depths 1.5 cm to 31.5 cm and field size $20\;{\text{cm}}^{2}$. The arrows show areas of mismatch.

Comparisons between TPS and IC diagonal profiles at depths 1.5 cm to 31.5 cm for field size $20\;{\text{cm}}^{2}$ are presented in Fig. 4. Only one algorithm was shown for each TPS, for brevity. XiO Superposition and IC profiles matched well at all depths. However, Eclipse AXB profiles were found to be higher than IC measured profiles at the shoulders at depths more than 11.5 cm. The mismatched worsened as depth increased from 21.5 cm to 31.5 cm. Monaco MC profiles also showed mismatch at depths 21.5 cm and 31.5 cm but, unlike Eclipse AXB, the disagreements were seen nearer to the center of the profiles, and the values computed by Monaco MC were lower than the IC measured profiles. The mismatches mentioned are indicated by arrows in Fig. 4 and quantified using absolute gamma analysis in Results section D below.

### C. Validation of IC profiles

**Figure 5:**
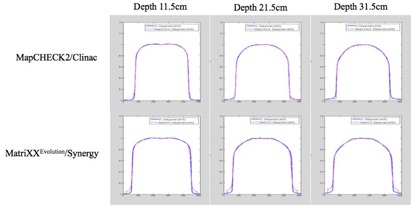
Diagonal relative profiles comparison between IC (blue solid line) and 2D array device (magenta dotted line). Good agreement was seen at all depths investigated.

The IC profiles at the diagonal axis were extracted from 2D dose planes created by simple cross‐multiplication of cross/in‐line IC profiles measured in a scanning water tank. Since the IC diagonal profiles were used as a reference, they were independently verified by comparing the IC diagonal profiles with profiles measured with commercial 2D array device. Figure 5 shows the diagonal profiles comparison between IC and 2D array, MapCHECK2 and ${\text{MatriXX}}^{\text{Evolution}}$, for Inst. A and Inst. B, respectively. Excellent agreements were seen for all depths, confirming the validity of the method used to create the IC 2D dose planes, as well as confirming the negligible effect of backscatter thickness on relative profiles.

### D. 2D absolute dose analysis

**Figure 6:**
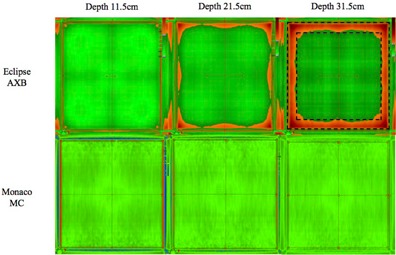
2D absolute gamma comparisons between TPS‐computed and IC‐measured dose planes for field size $20\;{\text{cm}}^{2}$. Areas that passed gamma criterion 3%/3 mm are indicated in green and areas of failure are indicated in red. The outer dashed box and the inner dashed box mark the 100% and 80% field areas included for analysis, respectively.

The relative profiles mismatched at depths more than 11.5 cm for Eclipse AXB and Monaco MC were evaluated quantitatively using 2D absolute gamma comparisons between TPS‐computed and IC‐measured dose planes (Fig. 6). Table 2 shows the percentage points that passed gamma criterion of 3%/3 mm in the whole field and at the center 80% of the fields. Overall, the gamma pass rates were better for Monaco MC than for Eclipse AXB. Monaco MC pass rates were 100% for all depths and field sizes investigated, while Eclipse AXB gamma pass rates ranged from 73.7% to 100%, with poorer pass rates as depth and field size increased. When only the center 80% of the field was analyzed, the Eclipse AXB gamma pass rates improved to above 98.1%.

**Table 2:** Results for 2D absolute gamma comparisons between TPS‐computed and IC‐measured dose planes

*Depth (cm)*	*Field Size* (${\text{cm}}^{2}$)	*Central Axis Point Dose* ^a^ *Difference*	*Gamma Criteria* (3%/3 mm)	
			*100% of field area*	*80% of field area*
*Eclipse AXB*				
11.5	10×10	1.2%	100.0%	100.0%
	20×20	0.9%	99.8%	100.0%
21.5	10×10	1.0%	98.2%	100.0%
	20×20	1.7%	89.2%	100.0%
31.5	10×10	1.6%	93.7%	98.4%
	20×20	2.3%	73.7%	98.1%
*Monaco MC*				
11.5	10×10	0.0%	100.0%	100.0%
	20×20	0.2%	100.0%	100.0%
21.5	10×10	0.9%	100.0%	100.0%
	20×20	1.3%	100.0%	100.0%
31.5	10×10	2.3%	100.0%	100.0%
	20×20	1.7%	100.0%	100.0%

a Central axis point dose difference=(TPS computed/IC measured)−1

## IV. DISCUSSION

### A. Consistency of data from different institutions

This study compared TPS computed doses with measured doses in two different institutions with TPS, linear accelerators, and dosimetry equipments of different models. To ensure that results were not affected by these differences, quality of TPS beam models and the methods used to collect data for this study must remain consistent in both institutions.

Commissioning data for all TPSs in both institutions were measured according to manufacturers' requirements. Similarly, beams were modeled according to manufacturers' methods, with no additional user intervention for the Eclipse and Monaco TPSs. Although XiO TPS beams were modeled in‐house, the procedures were also consistent with manufacturer's requirements. Therefore, any variation would be TPS/manufacturer‐specific and not due to differences introduced by users. In general, the manufacturers state an accuracy of better than 3% for the beam models. This specification was independently verified for each of the beam models by comparing the measured and modeled PDD curves for all the field sizes investigated in this study ($5\;{\text{cm}}^{2}$ to $20\;{\text{cm}}^{2}$). The measured PDD curves referred to the PDD values measured with an IC in a scanning water tank. The modeled PDD curves were represented by normalized doses computed with the beam models at different depths in a virtual water tank (dimension dimension 65 cm×65 cm×50 cm). By comparing the two curves, accuracy of the beam models can be quantified. Comparisons of values at different points along the PDD curves, up to depth 31.5 cm, showed an accuracy of better than 2% for all the beam models tested in this study. The average percentage differences were 0.4%±0.44% (Eclipse PBC), 0.6%±0.57% (Eclipse AAA), 0.8%±0.75% (Eclipse AXB), −0.4%±0.41% (XiO Convolution), −0.4%±0.67% (XiO Superposition), and A. 2%±0.50% (Monaco MC). This validation was important to ensure that baseline differences in beam models were not large enough to affect the outcome of this study.

As for the consistency in TPS dose calculations on virtual phantoms, the same settings, as far as possible, were used for all calculations. The same investigator created the virtual phantoms in all TPSs to avoid interpersonal variation. As these virtual phantoms were simple cubes with assigned uniform water density, the uncertainties related to inaccuracies of virtual phantoms creation were negligible. Lastly, differences in dosimetry equipment's model in the two institutions were mitigated using a ‘relative’ measurement technique. Readings were first taken in charge mode and later converted to dose using factor derived from measurements under calibration condition.

### B. central axis point dose deviation and BCF

Exit doses computed by Eclipse PBC and Eclipse AAA were found to be higher than IC measured doses by up to 5% (Figs. 2 and 3). To further investigate the large deviation seen with Eclipse PBC and Eclipse AAA, virtual scanning water tank phantom with dimension 65 cm×65 cm×50 cm was used to simulate full scatter condition. Exit doses were compared with doses calculated with full backscatter condition. In theory, the dose at the exit should be lower because of reduced backscattered photon. However, results from Eclipse PBC and Eclipse AAA showed similar doses for both conditions, with and without full backscatter. The average percentage dose difference was 0.4%±0.29% and 0.2%±0.20% for Eclipse PBC and Eclipse AAA, respectively. Furthermore, comparisons between Eclipse PBC and Eclipse AAA exit doses with doses calculated from PDD using Eq. (2) where full backscatter existed, again showed very similar values, with an average difference of 0.5%±0.41% and 0.4%±0.44% for Eclipse PBC and Eclipse AAA, respectively. This suggested that both algorithms do not consider the lack of backscatter and assume that full backscatter exists even at the exit level. This omission caused the dose at the exit level to be overestimated by the TPS as indicated in the comparison with IC measured dose. In clinical treatment planning, an error of 5% in the calculated exit dose, where the dose is originally low, would be of limited implication. However, this shortcoming in the TPS algorithms must be taken into account, especially in the case where *in vivo* exit dose measurements were directly compared to the TPS‐calculated values to avoid false‐positive results. This correction factor was tabulated in Table 1 as backscatter correction factor (BCF).

BCF values were almost identical for the two 6 MV beams with very similar beam quality investigated in this study. BCF is influenced by beam energy because the probability of Compton interaction and the direction of scatter are energy dependent. The effect of backscatter is less for higher energy beam due to more forward scatter and less large‐angle scatter photons. For the entrance BCF, the effect of backscatter contribution was as much as 2% to 3% (for backscatter thickness 1.5 cm and field size $20\;{\text{cm}}^{2}$). However, the extremely small backscatter thickness is not common in clinical practice. In this study, the dose reduction became negligible when thickness of underlying material was equal or more than 11.5 cm. For the same field size and depth, Hu and Zhu.^(29)^ reported a smaller thickness, 5 cm, to reach full backscatter condition. As for the field size influence on BCF, the backscatter effect is more prominent for bigger field size due to the larger area of scattering. For the exit BCF, the lack of backscattered photons at the exit level resulted in an approximately 4% reduction in dose at depth 31.5 cm and field size $20\;{\text{cm}}^{2}$. This value was within the ICRU recommendation of less than 5%^(30)^ and agreed favorably with published data. For example, at depth 20 cm and field size $20\;{\text{cm}}^{2}$, Kappas and Rosenwald^(31)^ reported a value of 0.967, compared to our value of 0.969 (Clinac) and 0.965 (Synergy). The backscattering effect was more obvious at larger depth because as the beam transverses the medium, the change in the beam spectrum results in higher relative contributions of backscattered photons. Finally, BCF values were found to be very similar between fixed SSD 100 cm and variable SSD. Since the fractional scatter contribution to depth dose is independent of the beam divergence,^(32)^ we would expect the backscatter effect to be independent, as well. Our results confirmed that the BCF table was independent of SSD and the same was reported by Lambert et al.^(33)^

### C. 1D relative profile and 2D absolute dose analysis

Relative profiles mismatched observed with Eclipse AXB and Monaco MC at depths more than 11.5 cm were quantified using 2D absolute gamma analysis, shown in Fig. 6. The Eclipse AXB profiles mismatched at the shoulders caused gamma failure at the field borders, which explained the improvement in gamma pass rate when only the center 80% of the field area was analyzed. Meanwhile, mismatched for Monaco MC profiles, which were lower than IC profiles near the center of the fields, were cancelled by the higher central axis point dose calculated by Monaco MC. This resulted in good gamma agreement using the 3%/3 mm gamma criteria.

## V. CONCLUSIONS

All six of the TPS algorithms accurately computed the central axis entrance doses to within 2%. For the exit doses, Eclipse PBC and Eclipse AAA algorithms failed to account for the lack of backscatter at the exit level, which resulted in central axis dose errors of up to 5%. Since the tolerance level for *in vivo* dosimetry is commonly set at 5%, the dose error for Eclipse PBC and Eclipse AAA must be considered to avoid false‐positive results. All other algorithms showed good agreement with IC measured, except for Eclipse AXB at depths more than 11.5 cm where 2D absolute gamma analysis failed mostly at the outer 20% of the field area. Accurate calculation of entrance and exit dose is essential if a TPS is to be used for reference dose comparisons in *in vivo* dosimetry. The findings of this study, therefore, serve as a useful baseline for the implementation of entrance and exit *in vivo* dosimetry in clinical departments utilizing any of these six common TPS algorithms for reference comparison.

## Supporting information

Supplementary Material

Supplementary Material
