# Supplementary figures and images for: Evaluation of six TPS algorithms in computing entrance and exit doses
Source: J Appl Clin Med Phys. 2014 May 8;15(3):229–40. doi: 10.1120/jacmp.v15i3.4739 (PMC5711058; doi:10.1120/jacmp.v15i3.4739)

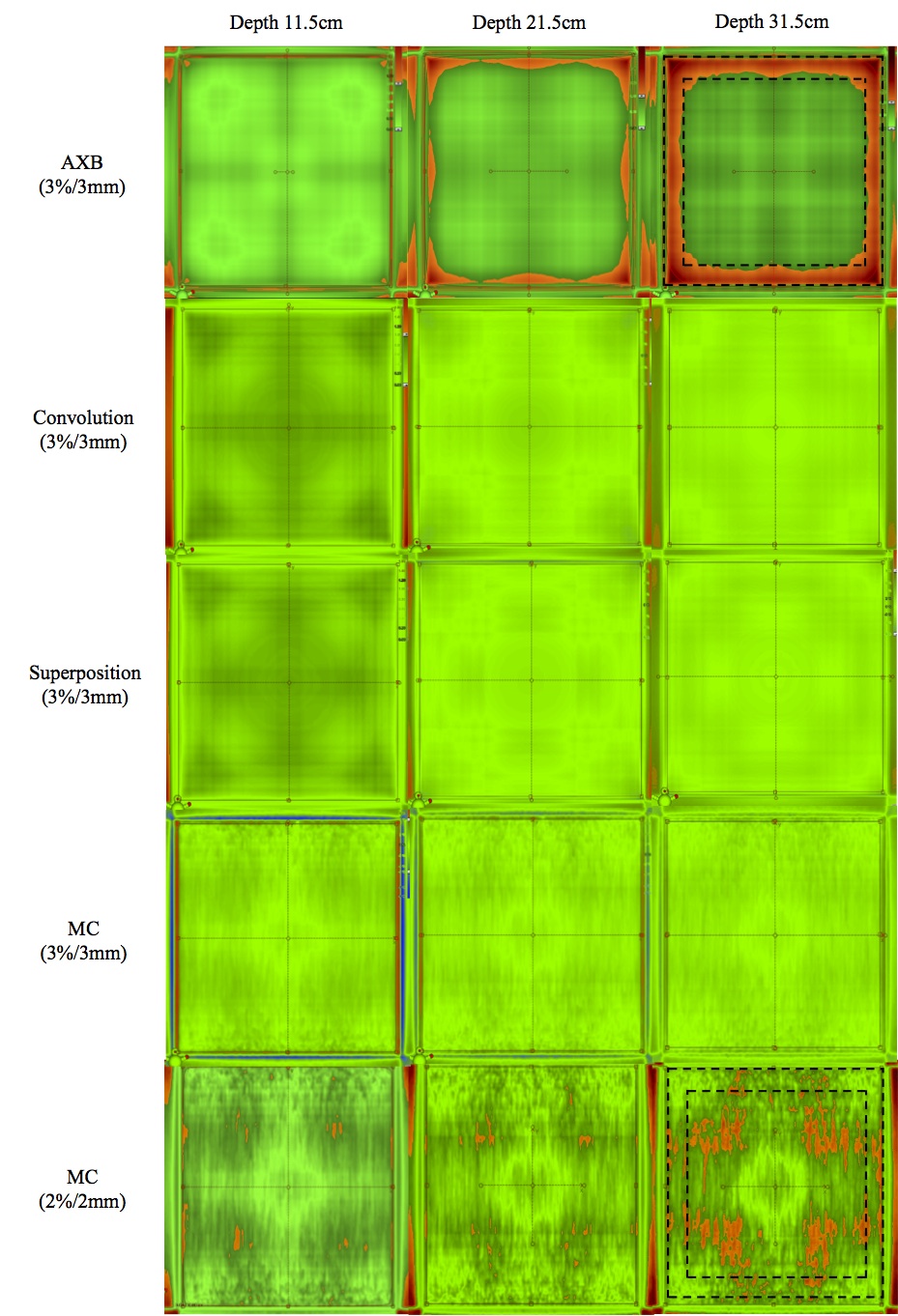

Supplement: Supplementary file 1 — Supplementary Material [file ACM2-15-229-s001.jpg]

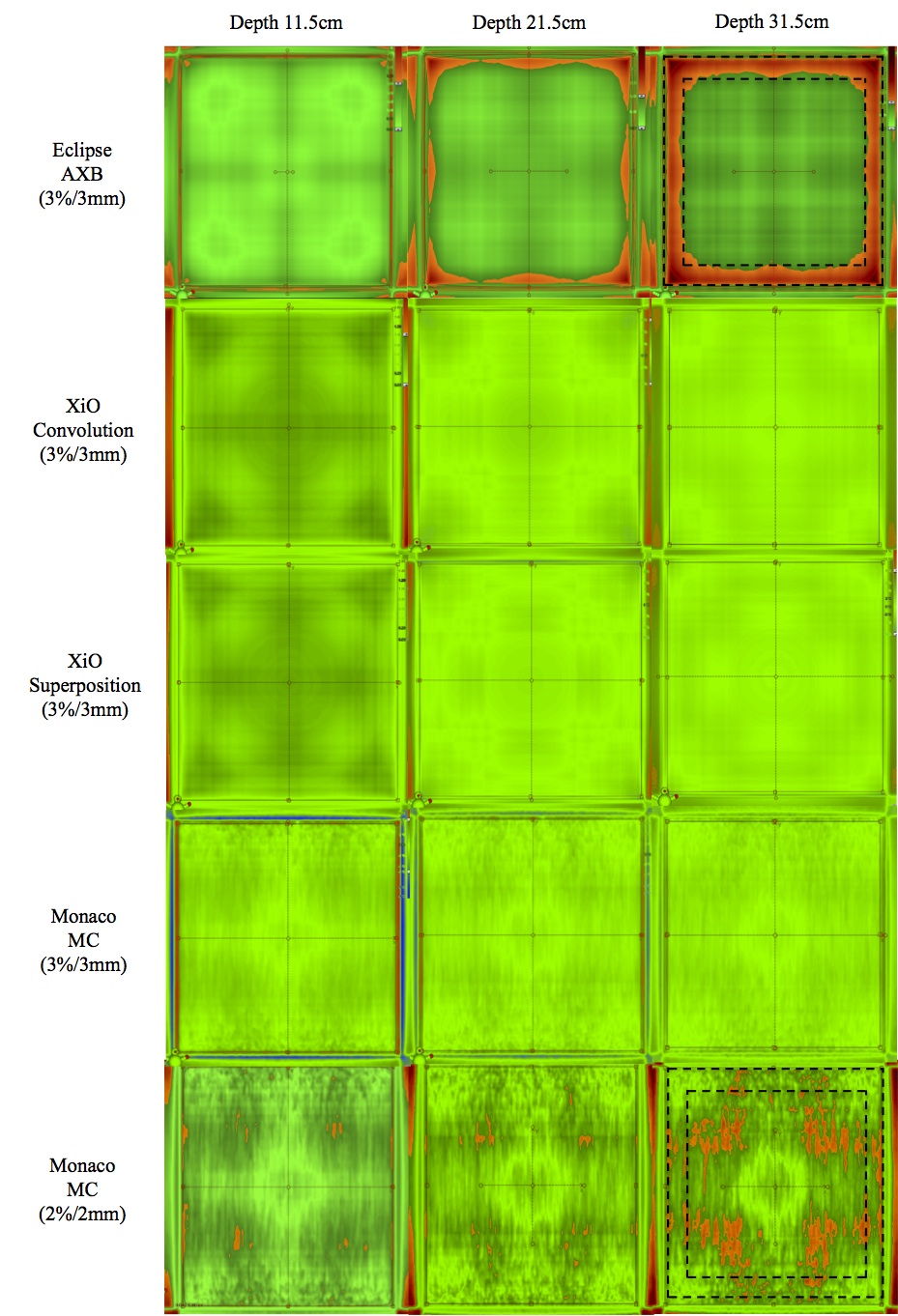

Supplement: Supplementary file 2 — Supplementary Material [file ACM2-15-229-s002.jpg]
